# Phage Morphology Recapitulates Phylogeny: The Comparative Genomics of a New Group of Myoviruses

**DOI:** 10.1371/journal.pone.0040102

**Published:** 2012-07-06

**Authors:** André M. Comeau, Denise Tremblay, Sylvain Moineau, Thomas Rattei, Alla I. Kushkina, Fedor I. Tovkach, Henry M. Krisch, Hans-Wolfgang Ackermann

**Affiliations:** 1 Québec-Océan, Département de Biologie, and Institut de Biologie Intégrative et des Systèmes (IBIS), Université Laval, Québec, Québec, Canada; 2 Félix d’Hérelle Reference Center for Bacterial Viruses, Groupe de Recherche en Écologie Buccale, Faculté de Médecine Dentaire, Université Laval, Québec, Québec, Canada; 3 Département de Biochimie, Microbiologie et Bio-Informatique, Faculté des Sciences et de Génie, Université Laval, Québec, Québec, Canada; 4 Department of Computational Systems Biology, University of Vienna, Vienna, Austria; 5 D.K. Zabolotny Institute of Microbiology and Virology, Kiev, Ukraine; 6 Centre National de Recherche Scientifique, Laboratoire de Microbiologie et Génétique Moléculaires, Toulouse, France; 7 Université de Toulouse-Paul-Sabatier, Laboratoire de Microbiologie et Génétique Moléculaires, Toulouse, France; 8 Département de Microbiologie-Infectiologie et Immunologie, Faculté de Médecine, Université Laval, Québec, Québec, Canada; Hospital for Sick Children, Canada

## Abstract

Among dsDNA tailed bacteriophages (*Caudovirales*), members of the *Myoviridae* family have the most sophisticated virion design that includes a complex contractile tail structure. The *Myoviridae* generally have larger genomes than the other phage families. Relatively few “dwarf” myoviruses, those with a genome size of less than 50 kb such as those of the Mu group, have been analyzed *in extenso*. Here we report on the genome sequencing and morphological characterization of a new group of such phages that infect a diverse range of *Proteobacteria*, namely *Aeromonas salmonicida* phage 56, *Vibrio cholerae* phages 138 and CP-T1, *Bdellovibrio* phage φ1422, and *Pectobacterium carotovorum* phage ZF40. This group of dwarf myoviruses shares an identical virion morphology, characterized by usually short contractile tails, and have genome sizes of approximately 45 kb. Although their genome sequences are variable in their lysogeny, replication, and host adaption modules, presumably reflecting differing lifestyles and hosts, their structural and morphogenesis modules have been evolutionarily constrained by their virion morphology. Comparative genomic analysis reveals that these phages, along with related prophage genomes, form a new coherent group within the *Myoviridae*. The results presented in this communication support the hypothesis that the diversity of phages may be more structured than generally believed and that the innumerable phages in the biosphere all belong to discrete lineages or families.

## Introduction

As all viruses, phages are classified by the International Committee on Taxonomy of Viruses according to their morphology and nucleic acid composition. The double-stranded DNA tailed phages, or *Caudovirales*, account for 96% of all the phages observed [1] and they belong to three families, *Myoviridae*, *Siphoviridae*, and *Podoviridae*. Members of the *Myoviridae*, such as the classical and well-studied phage T4, have a characteristic contractile tail structure. The myoviruses are currently further divided into 3 subfamilies, namely *Peduovirinae*, *Spounavirinae*, and *Tevenvirinae*; each of these subfamilies contains two genera. There are 11 other genera within the *Myoviridae* recognized by the ICTV [2], but these have not yet been assigned to subfamilies. The overwhelming majority of myoviruses remain unclassified because of insufficient data.

Recently, we have described two unassigned small myoviruses that were isolated on the little-studied gram-negative bacterial genera *Iodobacter* and *Bdellovibrio* (**Table 1**). The Iodobacteriophage φPLPE had a 47.5 kb genome, only about a quarter of the size of phage T4 whose genome size is 168 kb [3]. Numerous phages with virion dimensions similar to those of φPLPE (isometric heads of 60–70 nm and with 65–85 nm contractile tails) have been isolated on hosts from diverse bacterial genera such as *Aeromonas*, *Bdellovibrio*, *Bordetella*, *Pectobacterium*, *Vibrio*, and *Yersinia* belonging to the β, γ and δ branches of the *Proteobacteria*. Such phages of the φPLPE-type are morphologically indistinguishable and, hence, they could constitute a widespread set of phylogenetically related myoviruses.

**Table 1:** Known small myoviruses with isometric capsids.

		Dimensions (nm)				
Host	Phages^a^	Head	Tail	Source	Particulars	References
*Aeromonas salmonicida*	51, **56**, 57, 60	61	81×17	Freshwater, France	Typing phages	[31], [32]
*Aggregatibacter actinomycetemcomitans*	**Aaφ23**, Aaφ76, Aaφ97, AaφA99	68	112	Switzerland	Temperate	[4], [33]
	Aaφ247	60	115	Switzerland	Temperate	[34]
*Bdellovibrio bacteriovorus*	**φ1422**	68×40	62×12	Sewage, USA	Lytic	This work
	HDC-2	70–75	85?	Sewage, USA	?	[35]
	VL-1	60	80	Sewage, Israel	?	[36]
*Bordetella avium*	Ba1	55	85×14	USA	Temperate	[37]
	φATCC	63	80	USA	Temperate	–
*Bordetella parapertussis*	L1	63	80	Canada	Temperate	–
*Iodobacter sp.*	**φPLPE**	70	70×18	Freshwater, France	Lytic	[3]
*Pectobacterium carotovorum*	**ZF40**	58	86×15	Ukraine	Temperate	[7]
*Vibrio cholerae*	**CP-T1**	60^b^	65×10	Australia	Temperate	[38]
	**138**	63–66	∼81×17	Freshwater, India	Typing phage	[39], [40]
	13, 16, 24	63	84×17	England?	Typing phages	[39]
*Vibrio* sp.	O6N-21P, O6N-69P,O6N-86P	60–70	65–75×20	Seawater, Japan	Lytic	[41]
*Yersinia enterocolitica*	**PY100**	70	80	Pig manure, Germany	Lytic	[5]

a The genomes of the phages listed in bold are sequenced.

b A size of ∼45 nm was originally reported, but after our calibrated reexamination was found to be ∼60 nm.

A few morphologically similar phages, but having longer tails, have also been reported in the genus *Aggregatibacter*. One of the latter (Aaφ23) and a similar *Yersinia* phage (PY100) had been previously sequenced [4], [5]. By comparison, the smallest independent myovirus currently known is the Bdellovibriophage φ1402 [6], although comparable to the archetype T4 myovirus in having an elongated head, this phage’s virion’s dimensions are only a half of those of T4 and its 24 kb genome is merely a seventh of the size of T4’s and a half of that of φPLPE.

To continue our systematic analysis of small myoviruses, we decided to sequence and analyze the phylogenetic relationship of the diverse group of dwarf phages that all share a φPLPE-like virion morphology and are currently unassigned in the *Myoviridae* family.

## Methods

### Bacteria and Phages

*Aeromonas salmonicida* phage 56, *Vibrio cholerae* phages 138 (“group II”) and CP-T1, and their respective hosts are from the collection of the Félix d'Hérelle Reference Center for Bacterial Viruses (accession numbers HER 109, 52, and 373; www.phage.ulaval.ca). These phages were propagated for 3 h at 37°C in flasks containing 20 mL Trypticase Soy Broth and then filtered through 0.45 µm pore-size membranes. *Bdellovibrio* phage φ1422 was a gift from Dr. B. Fane from the University of Arizona at Tucson. *Pectobacterium carotovorum* (formerly *Erwinia carotovora*) phage ZF40 had been isolated and characterized in Kiev [7].

### Electron Microscopy

Phages 56, 138, CP-T1, and φ1422 were sedimented by centrifugation, washed, and stained as described earlier [6] and examined in a Philips EM 300 electron microscope using T4 tails as the magnification control.

### DNA Extraction and Sequencing

The DNAs of phages 56, 138, CP-T1, and φ1422 were extracted, precipitated and resuspended as described previously [6], [8]. The DNA of phage ZF40 was extracted in Kiev by a similar procedure. The resulting pure DNAs were used for bar-coded library construction and 454 pyrosequencing that was performed according to the manufacturer’s instructions on a quarter picotiter plate of a GS-FLX sequencer (Roche) at the IBIS/Université Laval Plate-forme d’Analyses Génomiques.

### Bioinformatics

Raw reads were assembled using the GS De Novo Assembler (Roche), resulting in one final contig for each phage with coverage ranging from 33- to 249-fold. Genome analysis was done with the following programs: 1) GLIMMER (www.ncbi.nlm.nih.gov/genomes/MICROBES/glimmer_3.cgi; >100 nt; bacterial genetic code) and GeneMark (exon.gatech.edu/GeneMark; heuristic approach for prokaryotes and viruses; >90 nt) for ORF determinations; 2) tRNA search using tRNAscan-SE (lowelab.ucsc.edu/tRNAscan-SE); 3) Java Word Frequencies (athena.bioc.uvic.ca) and Dot-Plot Alignments (MIPS Gepard; www.helmholtz-muenchen.de/en/mips/services/analysis-tools/gepard/index.html) for the exploration of DNA and protein “words”/patterns; 4) the BLAST tools at NCBI (blast.ncbi.nlm.nih.gov) for the characterization of genes/proteins and untranslated regions of the DNA; 5) various phylogenetic tools of the Mobyle Project at the Institut Pasteur (mobyle.pasteur.fr/cgi-bin/portal.py); 6) CoreGenes 3.1 (binf.gmu.edu:8080/CoreGenes3.1/) as described by Lavigne *et al.* [2], [9] for the count of shared proteins between the phages to the φPLPE reference genome; 7) we have developed our own program for evaluating the global level of sequence identity from MUMmer 3.22 alignments [10] which is available upon request; and 8) DNAPlotter for generating the circular genome visualizations (www.sanger.ac.uk/resources/software/dnaplotter). Statistical analyses (Mann-Whitney tests of non-normal similarity means) were carried out with PAST (http://folk.uio.no/ohammer/past/). The annotated phage genome sequences have been deposited in GenBank with the following accession numbers: JQ177061 (CP-T1 = vB_VchM-CP-T1), JQ177062 (φ1422 = vB_BbaM-phi1422), JQ177063 (phage 56 = vB_AsaM-56), JQ177064 (phage 138 = vB_VchM-138) and JQ177065 (ZF40 = vB_PcaM-ZF40).

**Figure 1:**
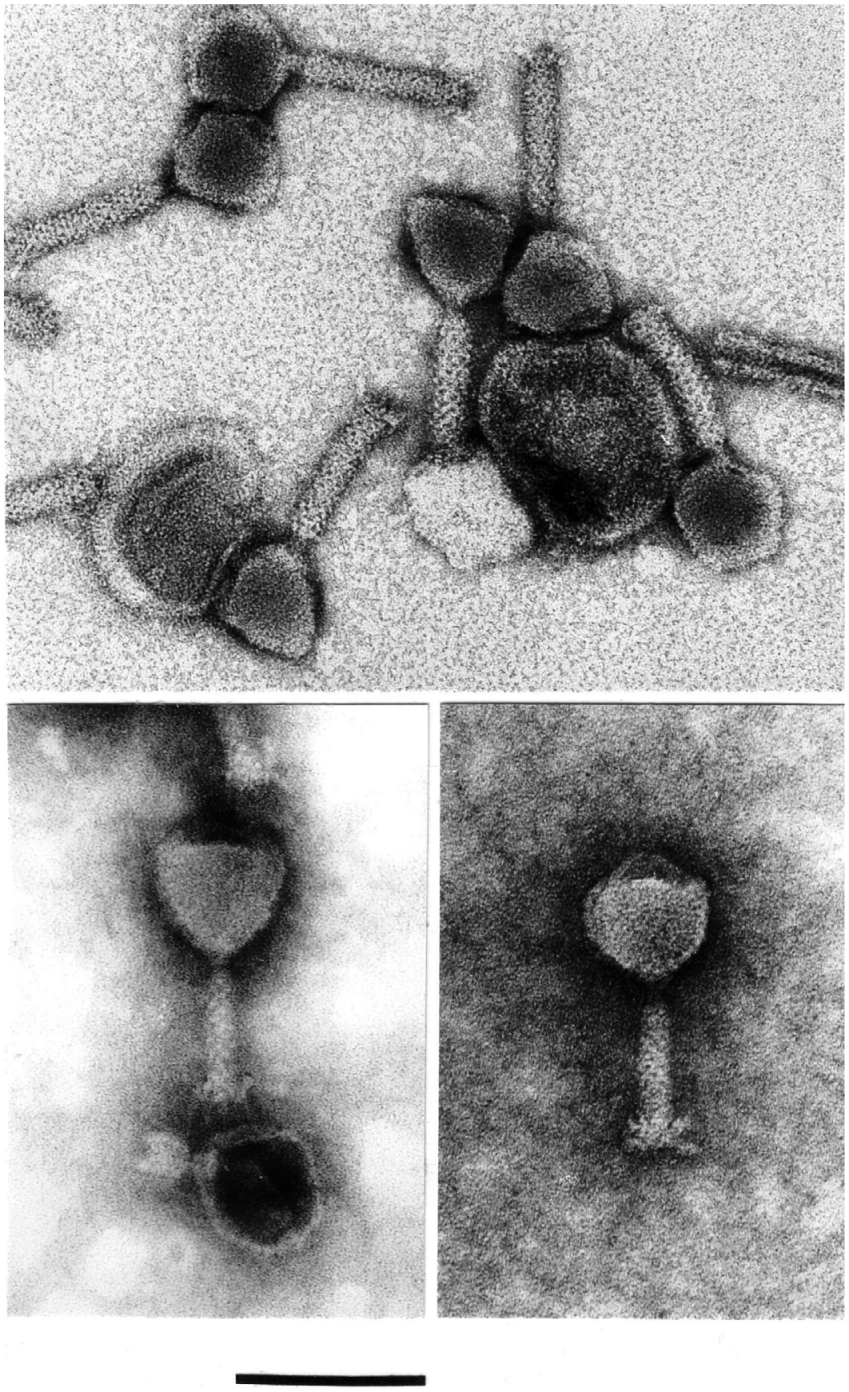
EM micrographs showing the morphology typical of the φPLPE group phages. Presented are *Aeromonas* phage 56 (top, uranyl acetate), *Bdellovibrio* phage φ1422 (bottom left, phosphotungstate) and *Vibrio* phage 138 (bottom right, phosphotungstate). The scale bar is 100 nm and applies to all micrographs.

**Table 2:** Characteristics of the five sequenced phage genomes compared to φPLPE, Aaφ23 and PY100.

				Phages genomes sequenced in this study				
	*Iodobacter*	*Aggregatibacter*	*Yersinia*	*Aeromonas*	*Bdellovibrio*	*Pectobacterium*	*Vibrio*	*Vibrio*
	φPLPE	Aaφ23	PY100	56	φ1422	ZF40	CP-T1	138
Characteristic	EU876853	AJ560763	AM076770	JQ177063	JQ177062	JQ177065	JQ177061	JQ177064
Genome size (bp)	47,453	43,033	50,291	43,551	45,354	48,454	44,492	44,485
%G+C content (range for host)	46 (50–52)	43 (44–45)	47 (47)	55 (58–59)	44 (51)	50 (52)	45 (47–48)	46 (47–48)
tRNAs	0	0	0	0	1	0	0	0
Observed lifestyle	Lytic	Temperate	Lytic	Lytic	Lytic	Temperate	Temperate	Lytic
*# ORFs:*	84	66	93	83	76	68	70	67
- Structure/morphogenesis	15	7	12	10	6	10	10	11
- Replication/recombination	6	7	8	8	5	8	7	8
- Lysis/lysogeny	3	10	1	2	–	7	–	1
- Unknown phage functions	13	35	15	21	9	21	26	24
- Cellular functions/hits	9	5	3	3	7	14	3	2
- ORFans	38 (45%)	2 (3%)	54 (58%)	39 (47%)	49 (64%)	8 (12%)	24 (34%)	21 (31%)
*“CoreGenes” shared with:*								
- φPLPE – over whole genome	84 (100%)	16 (19%)	14 (15%)	12 (14%)	9 (11%)	21 (25%)	29 (35%)	26 (31%)
- φPLPE – structural module only	47 (100%)	15 (32%)	12 (33%)	11 (23%)	9 (19%)	20 (43%)	28 (60%)	25 (53%)
*Presence of:*								
- Anti-repressor(s)	ant	ant/antB/cro	–	ant	–	cro	–	–
- Repressor(s) + Activator	–	cI	–	–	–	cI + cII	–	–
- Integrase(s) + Excisionase	–	√	–	–	–	int only	–	–
- Transposase	–	–	–	–	–	√	–	–
- Partition protein	–	–	–	–	√	–	–	–
- DNA polymerase	–	–	–	–	–	partial	√	√
- Helicase(s)	–	√	√	–	√	√	√	√
- Primase	–	–	–	–	√	–	√	√
- Single-stranded DNA binding protein	–	–	√	√	–	–	–	–
- DNA methylase(s)	–	√	√	√	–	√	√	√
- λ-like recombination proteins	√	√	–	√	–	–	–	√
- Holin/lysin + anti-holin	Holin/Lysin	√	Lysin	Lysin	Lysin	Holin/Lysin	–	Lysin
- Terminase – L and S subunits	√	√	√	√	L only	L only	√	√
- Quorum-sensing acylase	√	–	–	–	–	–	–	–
- Cellular transcriptional regulator	tetR	–	–	–	σ^54^	–	tetR	tetR

## Results and Discussion

### Morphology

The diverse phages presented in **Table 1** are morphologically related, but their capsids range in size from 55 to 75 nm and their tails from 62 to 115 nm. We have physically examined ourselves 14 of the phages in **Table 1**, namely 51, 56, 57, 60, φ1422, Bal, φATCC, L1, CP-T1, 138, 13, 16, 24 and ZF40. The virion morphology characteristic of the φPLPE-like phages is illustrated by the micrographs in **Figure 1**. For example, the *Aeromonas* phage 56, like *Iodobacter* phage φPLPE, has an icosahedral capsid of ≈61 nm, a contractile tail of ≈81×17 nm with no collar and short terminal fibers of about 10 nm in length. These dimensions are average values from over 150 observed virions. The tails of both phages exhibit faint cross striations or, less frequently, a criss-crossed pattern. The contracted sheaths measure about 37×20 nm and contraction separates the sheath from the base plate which then appears as a distinct thin disk of ≈17×2 nm (not shown). A few minor morphological variations do exist in the *Aggregatibacter* phages, such as Aaφ23, and in the *Bdellovibrio* phage φ1422 (**Table 1**). Although otherwise having identical dimensions to the other φPLPE-like phages, the former has a distinctly longer contractile tail structure of ≈112 nm and the latter has a slightly prolate capsid of 68×40 nm.

### Genome Analyses

**Figure 2:**
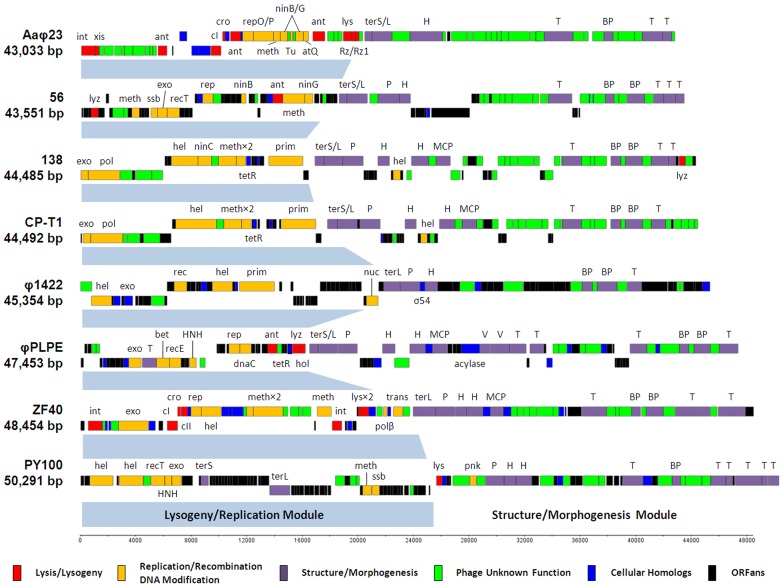
Comparative genomics of the eight φPLPE group phages. Note that the previously published sequences of φPLPE and PY100 have been re-cut to a similar organization as Aaφ23. Gene abbreviations/functions are as follows: acylase, homoserine lactone acylase; ant, anti-repressor; atQ, anti-termination; bet, lambda recombination; BP, baseplate; cI/II, repressor; cro, anti-repressor; dnaC, replication; exo, exonuclease; H, head; hel, helicase; HNH, HNH (homing) endonuclease; hol, holin; int, integrase; lys, lysis; lyz, lysozyme; MCP, major capsid protein; meth, methylase; ninB/C/G, lambda recombination; nuc, nuclease; P, portal; pnk, polynucleotide kinase; pol, DNA polymerase; prim, primase; rec(T), recombination; recE, exonuclease VIII; rep(O/P), (λ) replication; Rz/Rz1, lysis; ssb, single-stranded binding; σ^54^, bacterial transcriptional regulator; T, tail; terS/L, terminase; Tu, elongation factor; tetR, bacterial transcriptional regulator; trans, transposase; V, virion; xis, excisionase.

Among the phages of the φPLPE group that we have sequenced, they all are within the size-range of 43.5 to 48.5 kb, with phage 56 being the smallest and phage ZF40 the largest (**Table 2** and **Fig. 2**). All five genomes assembled as circular contigs indicating that they are circularly permuted and have direct terminal repeats [11]. GC contents ranged from 43–55% and most of them had only slightly lower GC levels than the genomes of their hosts; the only significant exception was the *Bdellovibrio* phage φ1422 which was seven percentage points lower, perhaps indicating a more recent phage-host association.

**Figure 3:**
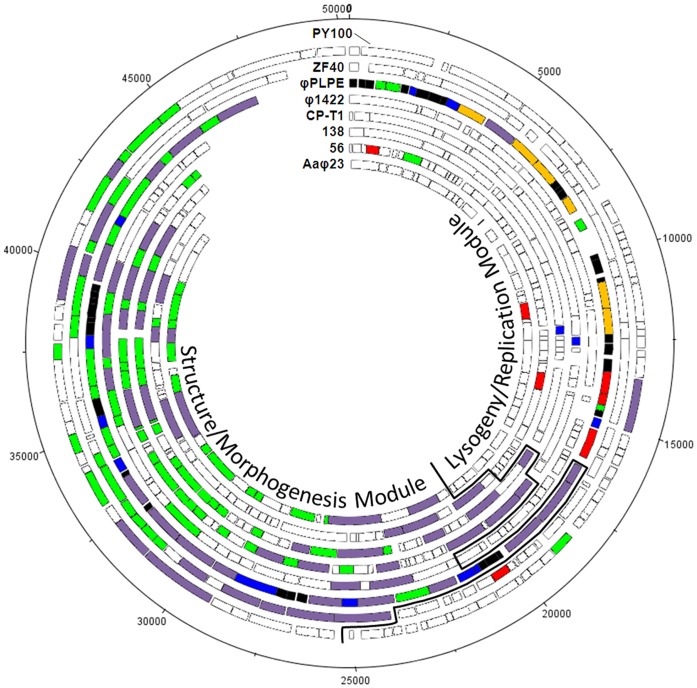
Bipartite nature of the φPLPE group phage genomes, with variable lysogeny/replication modules and conserved structure/morphogenesis modules. With the exception of φPLPE itself (all ORFs colored), only those ORFs shared with φPLPE in the other phages are color-coded as in Fig. 2. Shared ORFs were defined as protein matches in each phage against a φPLPE-restricted BLASTp with an *E*-value <10^−4^.

Curiously, the smallest phage genome actually had the most ORFs (>90 nt in size; consensus between GeneMark and GLIMMER). Perhaps less surprisingly, because of the limited number of *Bdellovibrio* phages that have been isolated and sequenced, the phage φ1422 has the largest number (64%) of ORFans (ORFs without known homologs). The atypically low numbers (3–8%) of ORFans in the genomes of phages ZF40 and Aaφ23 are the consequence of the presence of closely related prophages in the databases. Prophages similar to ZF40 are found in the bacterial genomes of *Yersinia frederiksenii* ATCC33641 (NZ_AALE00000000.2) and *Y. pseudotuberculosis* IP31758 (NC_009708.1). Similarly, the prophage S1249 integrated within the *A. actinomycetemcomitans* strain D11S-1 genome [12] has a large segment of homology to the Aaφ23 genome. There is also a prophage closely related to *Aeromonas* phage 56 within the *Oxalobacter formigenes* HOxBLS genome (NZ_ACDP00000000.1), but its homology is largely restricted to the right half of the genome, whereas the sequences of the prophages most closely related to ZF40 and Aaφ23 are distributed across their entire genomes. **Figure 3** reveals that the overall genome organization of φPLPE-type of phages is relatively well conserved.

**Table 3:** Repeat regions in the φPLPE-like phages.

Phage	Repeat Location	Repeat Size	Repeat Sequence
Iodobacteriophage φPLPE	46,046–46,255	210 bp	(KMGCCG)_35_
Vibriophage CP-T1	12,969–13,158	190 bp	(GCARACCTAYRCGRC)_12_GCAGACCTAC
Vibriophage 138	5,317–5,484	168 bp	(GGCGGCGGTGGYGTATCATCCTGCGGTTCRSTGTCAACAGGT)_4_

### Gene and Protein Functions

Considering their gene content, many of the φPLPE-like phages share similar protein functions in their lysogeny/replication modules (**Fig. 2** and **Table 2**), but these are often encoded by *analogs*, not *homologs,* and hence are not included among the shared ORFs compilation in **Figure 3**. Detailed annotations of the newly sequenced φPLPE-like phage genomes are presented in **Tables S1, S2, S3, S5, S5**.

**Figure 4:**
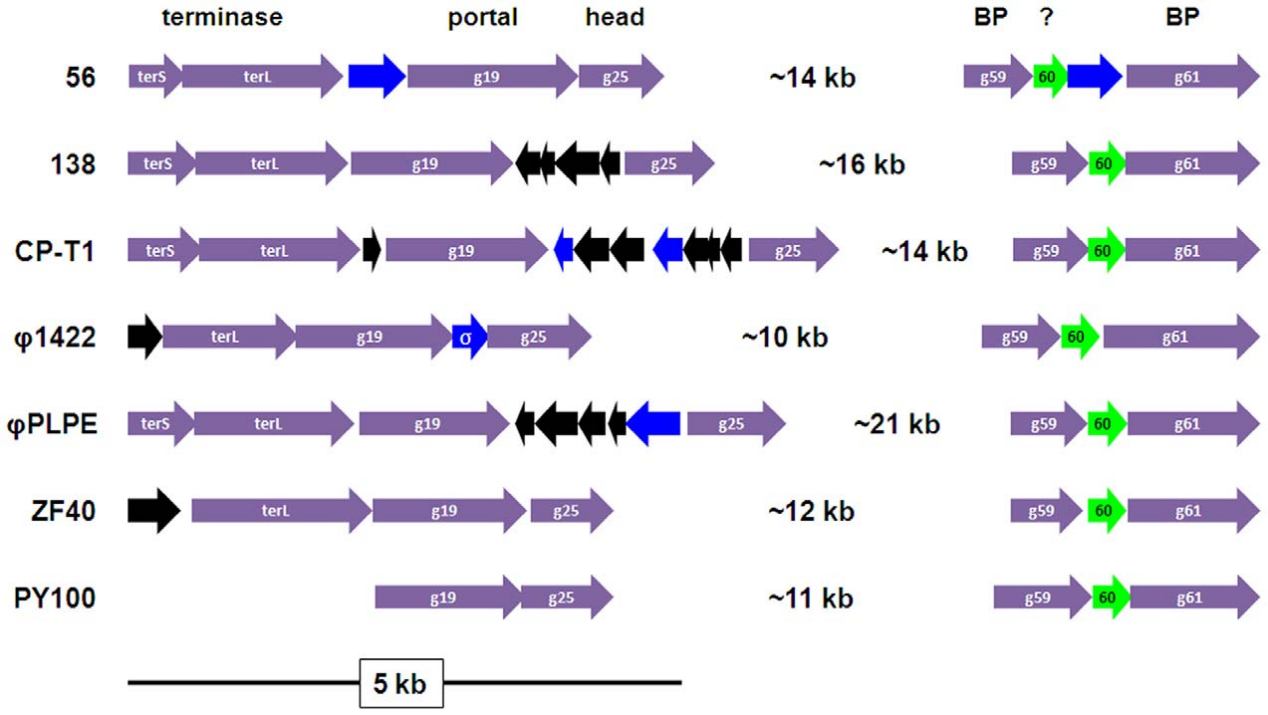
The two conserved structural mini-modules in the φPLPE group phages, excluding Aaφ23. Color-coding is as in Fig. 2 and gene numbers refer to the φPLPE genome. ORF60 (“60″) is of unknown function, but could be implicated in the baseplate (BP). The two small ORFs upstream of the *terL* genes in φ1422 and ZF40 could be the *terS* genes. The *terS/L* genes in PY100 are not arranged as in the other phages and are far upstream and not side-by-side. All of the cellular hits shown are (conserved) hypothetical bacterial proteins, except for φ1422 which has a σ^54^ transcription regulator (“σ”).

Some λ-like proteins involved in recombination and replication are shared by the φPLPE group; however, there are noteworthy exceptions. For example, the two vibriophages have B family DNA polymerases, whereas ZF40 has the β subunit of DNA polymerase I/II which, interestingly, is located upstream of a transposase. Three of the φPLPE-like phages (φPLPE, CP-T1 and 138) have tetR-like cellular transcriptional regulators, whereas φ1422 has a σ^54^-type regulator. Both types of regulators are implicated in bacterial response to osmotic stress [13], [14] and it is plausible that these bacterial genes have been co-opted by the phages for their own purposes. The trio of phages φPLPE, CP-T1 and 138 also share extended GC-rich repeat sequences (**Table 3**) that we speculated [3] could be a novel type of attachment site employed for lysogenic integration.

**Figure 5:**
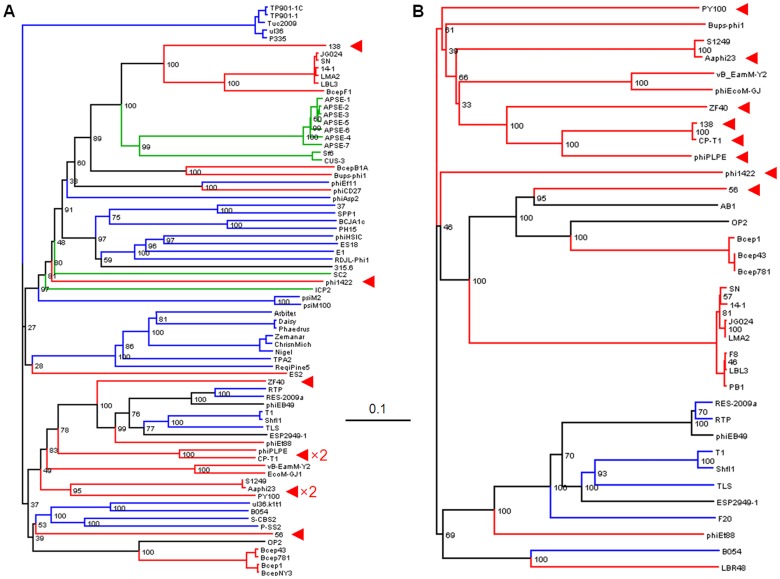
Neighbor-joining trees of TerL (A) and portal proteins (B; φPLPE gp19 homologs). The eight dwarf φPLPE-like myoviruses are highlighted with red arrows. Branches are colored according to phage family type: red for Myoviruses, blue for Siphoviruses, green for Podoviruses and black for unknown morphology. Values at the nodes are the results of 100 bootstrap replicates. The scale bar indicates 0.1 substitutions per site.

Considering their similar morphology, it is hardly surprising that the φPLPE-like phages all have a related structural-morphogenesis module (**Figs. 2** and **3**). The gene order of this structural module is well conserved: terminase – portal – head – tail – base-plate – tail fibers. There are two structural module subcomponents that are universally conserved in these phages (**Fig. 4**); one includes the terminase, portal and head proteins (with the exception of the placement of the *terS/L* in PY100). The other is near the end of the genome and most likely encodes the tail base-plate and tail fibers genes.

Finally, although many of the phages infect hosts that are human (*Vibrio*), plant (*Pectobacterium*) or animal (*Aeromonas*) pathogens, no identifiable toxins or virulence factors have been detected in any of these phage genomes.

**Figure 6:**
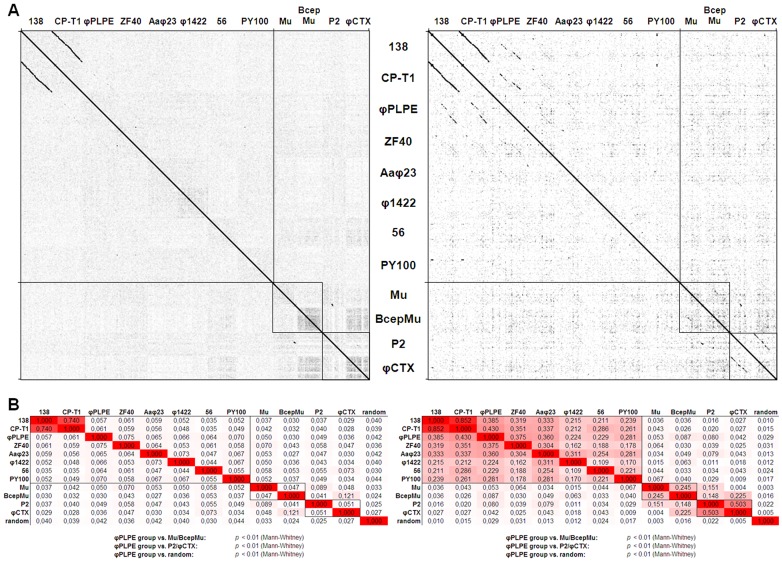
Whole genome similarities among φPLPE group phages. (**A**) Reciprocal dot-plots of the φPLPE-like phages based on whole genome nucleotide sequences (left) or concatenations of all proteins (right). Two Mu-like and two P2-like phages have been included for comparison. (**B**) Similarity matrices of the DNA sequences (left) and concatenated polyproteins (right) of the phages in (**A**). The non-φPLPE-like phages and a randomized sequence of φPLPE serve as controls. Also included are the results of the statistical tests comparing the similarity values of the φPLPE-like phages to the controls. Similarity values are highlighted with increasingly darker shades of red.

### Lytic vs. Temperate Lifestyles

All of the known temperate phages employ one of only three different systems for their lysogenic cycle: lambda-like integration/excision [15], Mu-like transposition [16] or plasmid-like partitioning of N15 [17]. With respect to the φPLPE group, their genomes possess a varying complement of lysogenic genes (**Fig. 2**, **Tables 1** and **2**). The phages Aaφ23 and ZF40 are known to be temperate and they have all of the classical lambda-like lysogeny genes (integrase, excisionase, repressor and antirepressor(s)). Moreover, the databases contain prophage sequences similar to both of these phages. Phage ZF40 only lacks an obvious excisionase, but these proteins are usually small, variable and can be difficult to identify by homology – perhaps one of the small proteins just downstream of the ZF40 integrase is a novel excisionase. Curiously, ZF40 also has a transposase, raising the possibility of a Mu-like transposition mechanism. Although this phage could have alternative lysogeny pathways, it seems more likely that the transposase was acquired by a random horizontal transfer and is not involved in lysogeny. Phage CP-T1 is the only other φPLPE-like phage known to be capable of temperate behavior [18], [19], but the genome has none of the genes required for any of the three known mechanisms of lysogeny mentioned above. Either this genome carries a novel set of genes that can assure a (pseudo-)lysogenic response that operates via a different mechanism than classical lysogeny or the Félix d’Hérelle Reference Center conserves a virulent mutant that has a spontaneous deletion of its lysogeny cassette. The phages PY100 and 138 are lytic phages and as expected their genomes carry none of the genes known to be involved in lysogeny. Phages 56 and φPLPE are also lytic, but both genomes contain a lambda-like anti-repressor sequence. This could either be the consequence of the random horizontal transfer of a lysogeny gene or, perhaps, the residue of a previously functional lysogeny cassette that has been largely deleted. Finally, the lytic φ1422 has a ParB homolog (gp42), implying either (as above) a horizontal acquisition or the possibility of a cryptic N15 plasmid-like partitioning system that has not manifested itself under the growth conditions we have employed.

### Phylogeny

In view of the strong morphological conservation among the φPLPE-like phages, it was somewhat surprising that their TerL large-subunit terminase sequences did not produce a simple and coherent phylogeny for the φPLPE group, suggesting instead that the group is polyphyletic **(** **Fig. 5A**). This non-structural gene, responsible for packaging the DNA into the capsid, has often been successfully employed as a phylogenetic marker gene for other phage groups [20], [21]. However, an often-used alternative marker, the portal protein that connects the phage capsid to the tail [22], [23], gave a much more coherent phylogeny, with the majority of the φPLPE-like phages forming a monophyletic group (**Fig. 5B**). It is perhaps relevant that while the terminase function is not a structural constituent of the virion, the portal protein is a central part of it.

To obtain a more global phylogenetic overview of the relationships between the different φPLPE phages, we have employed genomic dot-plots of these genomes sequences against each other (**Fig. 6A**). As controls, we have included representatives of the two other well-described groups of small myoviruses: P2 along with its close relative φCTX; and Mu along with its similarly close relative BcepMu. The dot-plot technique has been useful previously, especially to reveal weak sequence conservation between phage genomes that have diverged significantly from a distant common ancestor [24]. For example, for the large and extremely diverse T4 phage group, the virion structural module is visible as faint interrupted diagonal lines in plots of T4 against even some of the most distant members of this group [25]. This method has been successful in detecting distant phylogenetic relationships because both the sequence and synteny of virion structural genes are the most evolutionarily conserved features of phage genomes. Such genomic dot-plots reveal that only CP-T1 and 138, both replicating on the same host, share extended regions of nucleotide sequence homology. However, quantitative similarity analyses analysis of such data (**Fig. 6B**) clearly demonstrate that all the φPLPE-like phages are related and are significantly different (*p*<0.01) from either the Mu or P2 groups (similarities higher within the group than with the outsiders). Translating the genomic DNA sequences into a fusion polyprotein generally significantly improves detection levels due to the degeneracy of the genetic code. Consequently, the protein dot-plots reveal additional homologies among the members of the φPLPE group; for example, the broken diagonals for φPLPE (**Fig. 6A**). Nevertheless, this analysis of phages Aaφ23 through PY100 still does not reveal significant regions of substantial homology, having only a few homology “hotspots” here and there. In the control sequences, the pair Mu/BcepMu are fairly weak as well, whereas P2/φCTX show clearly visible diagonals. Our quantitative similarity analysis of the genome polyprotein sequences (**Fig. 6B**) convincingly demonstrates that the φPLPE-like phages are significantly different (*p*<0.01) from both the Mu and P2 groups. However, both Mu/BcepMu and P2/φCTX pairs show significant similarity within their respective groups, but there appears to have been some genetic blending between these two groups. These different types of polyprotein analyses are largely consistent with the conclusions of the BLAST analyses of the individual proteins presented in **Tables S1, S2, S3, S4, S5**.

### Conclusions

Dwarf myoviruses such as those described here have been much less studied from a genomic standpoint than their bigger cousins, with only two groups currently recognized on the NCBI Genome site: the P2-like phages (*Peduovirinae* [2]) representing 20 phage genomes of ≈31–41 kb and the Mu-like phages representing only three genomes of about 37 kb. Employing the approach Lavigne *et al.* [2], [9] used to update the *Myoviridae* and *Podoviridae* taxonomies, we tallied the shared protein sequences between the phages with a φPLPE-like morphology to the φPLPE reference genome (**Table 2**). The percentages of shared proteins over the entire genomes range from 11–35%; but, restricting the comparison to just the structure/morphogenesis modules and ignoring the more variable functions in the left-hand part of these genomes, the percentages essentially double to 19–60%. This data, coupled with our genome/protein similarity analyses (**Fig. 6B**), demonstrate that the φPLPE-like phages constitute a varied yet coherent set of phages that is clearly distinct from the other described myovirus types. This group’s unifying characteristics will probably become more evident and expand as additional related genomes are sequenced. Hopefully, for example, more details will emerge regarding the replisomes and lysis/lysogeny controls in these phages which are much more variable, perhaps to facilitate their adaptation to a wide variety of lifestyles, ecological niches and hosts.

Finally, these phages give us another example of a phylogenomic trend that is becoming increasingly evident as ever larger numbers of diverse phage genomes are sequenced – the core genomes of many groups seem to be built around a phylogenetically conserved virion module encoded by a coherent and largely fixed set of structural genes whose sequences have been mutually constrained during their evolution. We suggest that, as in the case of the T4 phage group, the φPLPE virion’s structural module has been subject to severe constraint of having to maintain a set of strong protein-protein interactions between the diverse virion components to insure a robust virion structure [26], [27]. Eons of Darwinian selection seem to have yielded only a limited number of successful virion structural modules that have the ability to easily adapt to new and varied ecological niches. This appears to have lead to an evolutionary scenario where the virion constituents have become relatively fixed while the other, mostly enzymatic, viral functions have been comparatively free to adapt to the requirements of their ever changing environment. One surprising direct consequence of this scenario has been that in spite of the enormous recent progress in phylogenomic analysis of phage diversity, a morphological classification seems to still be generally, although not perfectly, valid. For example, the divergent marine vibriophage VpV262 [28] and many cyanophages [29], [30] have host-derived DNA polymerases and photosynthesis genes, respectively, yet their virion morphology and the phylogenomics of their core genomes unambiguously place them within either the T7-like *Podoviridae* or the T4-like *Myoviridae*. One critical question for future studies to address is: how many phage morphotypes are there – a manageable number or a hopeless diversity of them? Our view is that this number is much smaller than would have been previously estimated and that consequently a coherent genomics-based phylogeny of the phage virosphere, guided by virion morphology, is now a feasible objective. Another important question for the phage genomics community to answer is: how much of phage evolution (and the taxonomy derived from it) is driven by the evolution of their structure vs. their (enzymatic/regulatory) function(s)?

## Supporting Information

Table S1 *Aeromonas* phage 56 ORFs with identifiable homologs/protein functions.
(PDF)

Table S2 *Bdellovibrio* phage φ1422 ORFs with identifiable homologs/protein functions.
(PDF)

Table S3 *Pectobacterium* phage ZF40 ORFs with identifiable homologs/protein functions.
(PDF)

Table S4 *Vibrio* phage CP-T1 ORFs with identifiable homologs/protein functions.
(PDF)

Table S5 *Vibrio* phage 138 ORFs with identifiable homologs/protein functions.
(PDF)

## References

[1] Ackermann H-W (2006). 5500 Phages examined in the electron microscope.. Arch Virol.

[2] Lavigne R, Darius P, Summer EJ, Seto D, Mahadevan P (2009). Classification of *Myoviridae* bacteriophages using protein sequence similarity.. BMC Microbiol.

[3] Leblanc C, Caumont-Sarcos A, Comeau AM, Krisch HM (2009). Isolation and genomic characterization of the first phage infecting *Iodobacteria*: φPLPE, a myovirus having a novel set of features.. Environ Microbiol Rep.

[4] Resch G, Kulik EM, Dietrich FS, Meyer J (2004). Complete genomic nucleotide sequence of the temperate bacteriophage Aaφ23 of *Actinobacillus actinomycetemcomitans*.. J Bacteriol.

[5] Schwudke D, Ergin A, Michael K, Volkmar S, Appel B (2008). Broad-host-range *Yersinia* phage PY100: Genome sequence, proteome analysis of virions, and DNA packaging strategy.. J Bacteriol.

[6] Ackermann H-W, Krisch HM, Comeau AM (2011). Morphology and genome sequence of phage φ1402: A dwarf myovirus of the predatory bacterium *Bdellovibrio bacteriovorus*.. Bacteriophage.

[7] Tovkach FI (2002). Temperate bacteriophage ZF40 of *Erwinia carotovora*: Phage particle structure and DNA restriction analysis.. Mikrobiologiya.

[8] Deveau H, van Calsteren MR, Moineau S (2002). Effect of exopolysaccharides on phage-host interactions in *Lactococcus lactis*.. Appl Environ Microbiol.

[9] Lavigne R, Seto D, Mahadevan P, Ackermann H-W, Kropinski AM (2008). Unifying classical and molecular taxonomic classification: Analysis of the *Podoviridae* using BLASTP-based tools.. Res Microbiol.

[10] Kurtz S, Phillippy A, Delcher AL, Smoot M, Shumway M (2004). Versatile and open software for comparing large genomes.. Genome Biol.

[11] Casjens SR, Gilcrease EB, Clokie MRJ, Kropinski AM (2009). Determining DNA packaging strategy by analysis of the termini of the chromosomes in tailed-bacteriophage virions..

[12] Chen C, Kittichotirat W, Si Y, Bumgarner R (2009). Genome sequence of *Aggregatibacter actinomycetemcomitans* serotype c strain D11S-1.. J Bacteriol.

[13] Ramos JL, Martínez-Bueno M, Molina-Henares AJ, Terán W, Watanabe K (2005). The TetR family of transcriptional regulators.. Microbiol Mol Biol Rev.

[14] Shingler V (2011). Signal sensory systems that impact σ^54^-dependent transcription.. FEMS Microbiol Rev.

[15] Oppenheim AB, Kobiler O, Stavans J, Court DL, Adhya S (2005). Switches in bacteriophage lambda development.. Ann Rev Genet.

[16] Chaconas G (1999). Studies on a “jumping gene machine”: Higher-order nucleoprotein complexes in Mu DNA transposition.. Biochem Cell Biol.

[17] Grigoriev PS, Lobocka MB (2001). Determinants of segregational stability of the linear plasmid-prophage N15 of *Escherichia coli*.. Mol Microbiol.

[18] Ogg JE, Shrestha MB, Poudayl L (1978). Phage-induced changes in *Vibrio cholerae*: Serotype and biotype conversions.. Infect Immun.

[19] Ogg JE, Timme TL, Alemohammad MM (1981). General transduction in *Vibrio cholerae*.. Infect Immun.

[20] Rao VB, Feiss M (2008). The bacteriophage DNA packaging motor.. Ann Rev Genet.

[21] Serwer P (2004). Improved isolation of undersampled bacteriophages: Finding of distant terminase genes.. Virol.

[22] Sullivan MB, Coleman ML, Quinlivan V, Rosenkrantz JE, Defrancesco AS (2008). Portal protein diversity and phage ecology.. Environ Microbiol.

[23] Olia AS, Prevelige Jr PE, Johnson JE, Cingolani G (2011). Three-dimensional structure of a viral genome-delivery portal vertex.. Nat Struct Mol Biol.

[24] Pope WH, Jacobs-Sera D, Russell DA, Peebles CL, Al-Atrache Z (2011). Expanding the diversity of mycobacteriophages: Insights into genome architecture and evolution.. PLoS ONE.

[25] Comeau AM, Bertrand C, Letarov A, Tétart F, Krisch HM (2007). Modular architecture of the T4 phage superfamily: A conserved core genome and a plastic periphery.. Virol.

[26] Häuser R, Sabri M, Moineau S, Uetz P (2011). The proteome and interactome of the *Streptococcus* phage Cp-1 virion.. J Bacteriol.

[27] Sabri M, Häuser R, Ouellette M, Liu J, Dehbi M (2011). Genome annotation and intra-viral interactome of the *Streptococcus pneumoniae* virulent phage Dp-1.. J Bacteriol.

[28] Hardies SC, Comeau AM, Serwer P, Suttle CA (2003). The complete sequence of marine bacteriophage VpV262 infecting *Vibrio parahaemolyticus* indicates that an ancestral component of a T7 viral supergroup is widespread in the marine environment.. Virol.

[29] Sullivan MB, Coleman ML, Weigele P, Rohwer F, Chisholm SW (2005). Three *Prochlorococcus* cyanophage genomes: Signature features and ecological interpretations.. PLoS Biol.

[30] Mann NH, Clokie MRJ, Millard A, Cook A, Wilson WH (2005). The genome of S-PM2, a “photosynthetic” T4-type bacteriophage that infects marine *Synechococcus* strains.. J Bacteriol.

[31] Ackermann H-W, Dauguet C, Paterson WD, Popoff M, Rouf MA (1985). *Aeromonas* bacteriophages: Reexamination and classification.. Ann Inst Pasteur Virol.

[32] Popoff M (1971). Étude sur les *Aeromonas salmonicida*. II. Caractérisation des bactériophages actifs sur les *Aeromonas salmonicida* et lysotypie.. Ann Rech Vet.

[33] Willi K, Sandmeier H, Meier J (1993). Temperate bacteriophages of *Actinobacillus actinomycetemcomitans* associated with periodontal disease are genetically related.. Med Microbiol Lett.

[34] Willi K, Sandmeier H, Asikainen S, Saareda M, Meyer J (1997). Occurrence of temperate bacteriophages in different *Actinobacillus actinomycetemcomitans* serotypes isolated from periodontally healthy individuals.. Oral Microbiol Immunol.

[35] Althauser M, Samsonoff WA, Anderson C, Conti SF (1972). Isolation and preliminary characterization of bacteriophages for *Bdellovibrio bacteriovorus*.. J Virol.

[36] Varon M, Levisohn R (1972). Three-membered parasitic system: A bacteriophage, *Bdellovibrio bacteriovorus*, and *Escherichia coli*.. J Virol.

[37] Shelton CB, Crosslin DR, Casey JL, Hg S, Temple LM (2000). Discovery, purification, and characterization of a temperate transducing bacteriophage for *Bordetella avium*.. J Bacteriol.

[38] Guidolin A, Morelli G, Kamke M, Manning PA (1984). *Vibrio cholerae* bacteriophage CP-T1: Characterization of bacteriophage DNA and restriction analysis.. J Virol.

[39] Ackermann H-W, Furniss AL, Kasatiya SS, Lee JV, Mbiguino A (1983). Morphology of *Vibrio cholerae* typing phages.. Ann Inst Pasteur Virol.

[40] Chatterjee SN, Das J, Barua D (1965). Electron microscopy of cholera phages.. Indian J Med Res.

[41] Hidaka T, Fujimura T (1971). A morphological study of marine bacteriophages.. Mem Fac Fish Kagoshima Univ.

