# Supplementary material for: Phage Morphology Recapitulates Phylogeny: The Comparative Genomics of a New Group of Myoviruses
Source: PLoS One. 2012 Jul 6;7(7):e40102. doi: 10.1371/journal.pone.0040102 (PMC3391216; doi:10.1371/journal.pone.0040102)
Supplement: Table S3 — Pectobacterium phage ZF40 ORFs with identifiable homologs/protein functions. (PDF) [file pone.0040102.s003.pdf]

Table S3. *Pectobacterium* phage ZF40 ORFs with identifiable homologs/protein functions.

ORF	Strand	Start	End	nt length	aa length	*E*-value	Homolog(s)/Function[a]
2	-	567	1592	1026	341	3e-101	Integrase protein [*Salmonella* phage ST64B]; many phage integrases
3	-	1595	1810	216	71	2e-9	gp29 [*Salmonella* phage ST64B]
4	-	1761	1967	207	68	5e-17	gp12720 [*Yersinia frederiksenii* ATCC 33641]
5	-	2014	2190	177	58	1e-16	gp4146 [*Salmonella enterica* str. CVM23701]
6	-	2223	2738	516	171	2e-23	gp46 protein [*Listeria* phage 2389]; P/BCHP
7	-	2735	4900	2166	721	5e-40	putative exonuclease [*Pseudomonas* phage F116]; many exonuclease VIII or RecE
8	-	4919	5407	489	162	2e-13	gp03953 [*Providencia alcalifaciens* DSM 30120]
10	-	6251	6994	744	247	1e-44	cI [Enterobacteria phage P22]; λ cI
11	+	6987	7193	207	68	4e-16	cro [Enterobacteria phage CUS-3]; λ cro
12	+	7249	7701	453	150	5e-10	cII [Enterobacteria phage ϕ80]; λ cII
13	+	7715	7939	225	74	2e-36	gp12630 [*Yersinia frederiksenii* ATCC 33641]; possible adenylosuccinate synthase
14	+	7957	8712	756	251	5e-46	replication protein [*Aggregatibacter* phage S1249]; many replication proteins
15	+	8702	10114	1413	470	2e-149	phage HK022 P protein [Enterobacteria phage VT2-Sakai]; many DnaB helicases
16	+	10157	10636	480	159	5e-24	gp42700 [*Yersinia frederiksenii* ATCC 33641]
17	+	10639	10893	255	84	8e-20	gp2823 [*Serratia* sp. AS12]
18	+	10890	11726	837	278	3e-10	gp37 [*Salmonella enterica* str. RI_05P066]
19	+	11723	11974	252	83	2e-10	gp49 [Enterobacteria phage Sf6]
20	+	11971	12447	477	158	1e-61	methyltransferase [*Listeria* phage A118]; many DNA N-4 cytosine methyltransferases
21	+	12444	14597	2154	717	4e-122	methyltransferase [*Pseudomonas* phage F116]; many DNA C-5 cytosine methyltransferases
22	+	14594	14941	348	115	2e-45	gp55 [Enterobacteria phage phiV10]; P/BCHP
23	+	15098	15691	594	197	7e-77	PCHP [Enterobacteria prophage Gifsy-1]
24	+	15688	15972	285	94	2e-46	gp136 [Enterobacteria phage ST64T]; P/BCHP
25	+	16004	16609	606	201	2e-65	gp49 [*Salmonella* phage ST64B]
27	+	17021	18055	1035	344	4e-97	Dam [Enterobacteria phage P7]
28	-	18109	18804	696	231	5e-57	integrase [Enterobacteria phage 21]; λ int
30	-	19220	19570	351	116	1e-27	gp0840 [*Photobacterium profundum* SS9]; BCHP
33	+	20000	20212	213	70	2e-31	lysis protein [Enterobacteria phage cdtI]; λ lysis protein S
34	+	20212	20742	531	176	5e-107	lysis protein [*Yersinia* phage PY54]; gp15 putative lysozyme [ϕPLPE]
35	+	20739	21263	525	174	4e-52	gp4986 [*Rahnella* sp. Y9602]; BCHP
36	+	21333	21740	408	135	9e-32	gp66 [*Burkholderia* phage ϕ1026b]; P/BCHP
37	+	21812	22066	255	84	1e-32	Hot [Enterobacteria phage P1]; DNA pol II or III
38	+	22120	22341	222	73	3e-10	Methyl-accepting chemotaxis protein [*Yersinia frederiksenii* ATCC 33641]

39	+	22509	23186	678	225	6e-109	putative transposase [*Cronobacter* phage ENT47670]; many transposases and P/BCHP
40	+	23219	23704	486	161	2e-66	gp30 [*Cronobacter* phage ENT47670]; PCHP
41	+	23938	25581	1644	547	1e-149	TerL [*Erwinia* phage ϕEt88]
42	+	25581	26975	1395	464	3e-134	gp19 putative portal protein [ϕPLPE]
43	+	27025	27774	750	249	6e-26	head protein [*Yersinia* phage PY100]; gp25 putative head protein [ϕPLPE]
44	+	27784	28971	1188	395	2e-84	gp27 putative head protein [ϕPLPE]
45	+	28971	29486	516	171	3e-67	gp2221 [*Yersinia pseudotuberculosis* IP 31758]
46	+	29502	30437	936	311	3e-18	gp29 major capsid protein [ϕPLPE]
47	+	30427	30975	549	182	3e-22	gp43060 [*Yersinia frederiksenii* ATCC 33641]
48	+	31005	31427	423	140	1e-21	gp41 [ϕPLPE]; PCHP
49	+	31424	31891	468	155	3e-15	gp07 [Deep-sea thermophilic phage D6E]; gp42 [ϕPLPE]; PCHP
50	+	31894	32316	423	140	1e-10	gp43 [ϕPLPE]
51	+	32316	32840	525	174	9e-10	gp44 [ϕPLPE]
52	+	32849	34003	1155	384	6e-61	gp46 [ϕPLPE]
53	+	34009	34413	405	134	3e-18	gp47 [ϕPLPE]
54	+	34413	34814	402	133	6e-53	gp43000 [*Yersinia frederiksenii* ATCC 33641]
55	+	34883	34984	102	33	2e-10	gp2215 [*Yersinia pseudotuberculosis* IP 31758]
57	+	36100	37860	1761	586	7e-22	putative muramidase [*Aeromonas* phage PAS-1]; C-term lysozyme, N-term tape measure
58	+	37863	38507	645	214	7e-17	gp56 [ϕPLPE]
59	+	38504	38794	291	96	4e-8	gp57 [ϕPLPE]
60	+	38787	39674	888	295	7e-15	gp58 [ϕPLPE]; PCHP
61	+	39674	40330	657	218	9e-26	gp59 putative baseplate protein [ϕPLPE]
62	+	40388	40738	351	116	5e-22	gp60 [ϕPLPE]; PCHP
63	+	40738	41940	1203	400	6e-69	gp61 putative baseplate protein [ϕPLPE]
64	+	41933	42808	876	291	6e-23	gp62 [ϕPLPE]; PCHP
65	+	42843	45332	2490	829	8e-14	gp173 [*Bathycoccus* sp. RCC1105 virus BpV2]; many P/B fiber proteins
66	+	45348	45845	498	165	1e-8	gp51 [*Azospirillum* phage Cd]
67	+	45906	47873	1968	655	8e-39	gp05 H [*Burkholderia* phage BcepB1A]; many P/B tail fibers

[a] Generally listed are the closest phage (if any) homologs of identifiable function, followed by identifiable bacterial homologs, and then phage/bacterial hypothetical proteins (if no function could be deduced) from BLASTp searches against the nr (or phage restricted) database using an *E*-value cutoff of $<10^{-4}$. Also included are additional hits/homologs of interest and hits to the Conserved Domain Database (CDD) with their cd/pfam/cl/PHA identifiers. (P/B)CHP = (phage/bacteria) conserved hypothetical protein.
