# Supplementary material for: Phage Morphology Recapitulates Phylogeny: The Comparative Genomics of a New Group of Myoviruses
Source: PLoS One. 2012 Jul 6;7(7):e40102. doi: 10.1371/journal.pone.0040102 (PMC3391216; doi:10.1371/journal.pone.0040102)
Supplement: Table S2 — Bdellovibrio phage φ1422 ORFs with identifiable homologs/protein functions. (PDF) [file pone.0040102.s002.pdf]

Table S2. *Bdellovibrio* phage ϕ1422 ORFs with identifiable homologs/protein functions.

ORF	Strand	Start	End	nt length	aa length	*E*-value	Homolog(s)/Function[a]
1	+	1	828	828	275	3e-46	gp39 [*Bdellovibrio* phage ϕ1402]
2	-	803	2251	1449	482	7e-56	putative helicase [Enterobacteria phage Eco1230-10]; COG1061 helicase
4	-	2390	2896	507	168	6e-8	gp01178 [*Bacteroides caccae* ATCC 43185]
6	-	3133	3777	645	214	1e-8	DEAD/DEAH box helicase domain-containing protein [*Desulfococcus oleovorans* Hxd3]; cd05782 DEDDy exonuclease
10	-	5105	6046	942	313	2e-29	gp14 [*Bacillus* phage BCJA1c]
13	+	6766	7680	915	304	6e-12	gp47 [*Listeria* phage A118]; cl09232 YqaJ viral recombinase
18	+	8699	9088	390	129	6e-6	gp51 [*Staphylococcus warneri* L37603]
20	+	9508	10944	1437	478	3e-20	PcrA helicase [*Bacillus* phage 0305ϕ8-36]; COG0210 UvrD helicase
22	+	11175	11366	192	63	3e-6	SecC motif-containing protein [*Methylobacterium radiotolerans* JCM 2831]
23	+	11480	13996	2517	838	2e-26	putative primase [*Stenotrophomonas* phage S1]; DNA primase traC
42	-	20579	21451	873	290	2e-12	ParB-like nuclease [*Riemerella* phage RAP44]
44	+	21819	23045	1227	408	7e-98	large terminase subunit [*Salmonella* phage E1]
45	+	23038	24474	1437	478	3e-21	gp19 putative portal protein [ϕPLPE]
46	+	24471	24800	330	109	2e-6	σ^{54} dependent transcrip. regulator [*Shewanella* sp. W3-18-1]; pfam02954 Fis regulator
47	+	24797	25753	957	318	2e-15	putative head protein [*Burkholderia* phage Bups ϕ 1]; gp25 putative head protein [ϕPLPE]
50	+	27322	28353	1032	343	4e-6	gp35 [Aaϕ23]
51	+	28422	28859	438	145	1e-5	putative bacteriophage protein [*Escherichia coli* F11]
55	+	30484	31899	1416	471	2e-17	gp39 [Aaϕ23]
62	+	35279	36148	870	289	6e-17	gp45 [Aaϕ23]
63	+	36141	36869	729	242	4e-16	gp3.3 [*Burkholderia* phage Bups ϕ1]; gp59 putative baseplate protein [ϕPLPE]
64	+	36866	37225	360	119	2e-13	gp47 [Aaϕ23]
65	+	37234	38658	1425	474	2e-23	gp2213 [*Aggregatibacter* phage S1249]; gp61 putative baseplate protein [ϕPLPE]
66	+	38658	39359	702	233	4e-14	gp2212 [*Aggregatibacter* phage S1249]
67	+	39369	40427	1059	352	2e-14	putative tail fiber protein [*Tsukamurella* phage TPA2]; gp35 virion protein [ϕPLPE]
71	+	42946	43173	228	75	1e-17	gp36 [*Bdellovibrio* phage ϕ1402]
72	+	43196	43435	240	79	2e-23	gp37 [*Bdellovibrio* phage ϕ1402]
76	+	44812	45345	534	177	2e-26	phage-related lysozyme [*Rhizobium* sp. NGR234]

[a] Generally listed are the closest phage (if any) homologs of identifiable function, followed by identifiable bacterial homologs, and then phage/bacterial hypothetical proteins (if no function could be deduced) from BLASTp searches against the nr (or phage restricted) database using an *E*-value cutoff of $<10^{-4}$. Also included are additional hits/homologs of interest and hits to the Conserved Domain Database (CDD) with their cd/pfam/cl/PHA identifiers. (P/B)CHP = (phage/bacteria) conserved hypothetical protein.
