# Supplementary material for: Phage Morphology Recapitulates Phylogeny: The Comparative Genomics of a New Group of Myoviruses
Source: PLoS One. 2012 Jul 6;7(7):e40102. doi: 10.1371/journal.pone.0040102 (PMC3391216; doi:10.1371/journal.pone.0040102)
Supplement: Table S4 — Vibrio phage CP-T1 ORFs with identifiable homologs/protein functions. (PDF) [file pone.0040102.s004.pdf]

Table S4. *Vibrio* phage CP-T1 ORFs with identifiable homologs/protein functions.

ORF	Strand	Start	End	nt length	aa length	*E*-value	Homolog(s)/Function[a]
2	-	189	722	534	177	9e-55	putative exodeoxyribonuclease VIII [*Salmonella* phage epsilon34]; PHA02570 DexA
3	-	727	3063	2337	778	6e-46	DNA polymerase [*Burkholderia* phage BcepGomr]; cd05538 phage DNA PolB
4	-	3060	3368	309	102	3e-8	gp12 [Enterobacteria phage N4]
5	-	3424	4308	885	294	2e-151	gp68 [*Burkholderia* phage BcepB1A]
7	-	4530	5711	1182	393	9e-34	gp67 [*Burkholderia* phage BcepB1A]; pfam10926 PCHP
10	+	6842	9823	2982	993	3e84	gp65 [*Burkholderia* phage BcepB1A]; COG1061 helicase
11	+	9793	10323	531	176	5e-38	gp62 [*Burkholderia* phage BcepB1A]
12	+	10320	11621	1302	433	8e-114	Dmt [Enterobacteria phage P1]; cd00315 DNA methylase
13	+	11618	12319	702	233	3e-96	adenine-specific DNA methyltransferase [*Pseudoalteromonas* phage H105/1]; pfam01555
14	+	12368	12673	306	101	1e-10	gp14 [ϕPLPE]; putative TetR family transcriptional regulator
17	+	13628	13831	204	67	7e-7	gp0660 [*Thiomicrospira crunogena* XCL-2]
20	+	14453	16933	2481	826	2e-40	gp49 primase [*Burkholderia* phage BcepB1A]
22	+	17808	18491	684	227	2e-19	TerS [ϕPLPE]
23	+	18481	19941	1461	486	0	TerL [ϕPLPE]
25	+	20113	21582	1470	489	0	gp19 putative portal protein [ϕPLPE]
26	-	21620	21802	183	60	2e-29	gp1938 [*Vibrio cholerae* 1587]
29	-	22495	22776	282	93	3e-42	gp226 [*Vibrio* phage ICP1]
33	+	23379	24200	822	273	7e-89	gp25 putative head protein [ϕPLPE]
35	-	24489	24992	504	167	4e-30	putative helicase [*Vibrio* phage VP16T]; COG1896 predicted hydrolase
37	-	25188	25496	309	102	3e-5	gp14 [ϕPLPE]
39	+	25855	26994	1140	379	1e-72	gp27 putative head protein [ϕPLPE]
40	+	26976	27500	525	174	2e-18	gp28 [ϕPLPE]; P/BCHP
41	+	27506	28519	1014	337	4e-96	gp29 major capsid protein [ϕPLPE]
42	+	28532	28963	432	143	4e-6	gp30 [ϕPLPE]
44	+	29648	30073	426	141	6e-34	gp41 [ϕPLPE]; P/BCHP
47	+	30680	31150	471	156	2e-25	gp42 [ϕPLPE]; P/BCHP
48	+	31151	31531	381	126	5e-30	gp43 [ϕPLPE]
49	+	31528	32064	537	178	2e-50	gp44 [ϕPLPE]
50	+	32097	33242	1146	381	2e-166	gp46 [ϕPLPE]; P/BCHP
51	+	33256	33669	414	137	4e-56	gp47 [ϕPLPE]
54	+	34126	34539	414	137	2e-9	gp48 [ϕPLPE]
55	+	34584	34706	123	40	1e-8	gp49 [ϕPLPE]
56	+	34708	36072	1365	454	3e-53	gp55 putative tail-fiber/lysozyme protein [ϕPLPE]

57	+	36082	36729	648	215	2e-72	gp56 [ϕPLPE]; P/BCHP
58	+	36726	37034	309	102	2e-25	gp57 [ϕPLPE]
59	+	37027	37881	855	284	8e-88	gp58 [ϕPLPE]; P/BCHP
60	+	38207	38905	699	232	3e-95	gp59 putative baseplate protein [ϕPLPE]
61	+	38902	39252	351	116	6e-49	gp60 [ϕPLPE]; P/BCHP
62	+	39245	40468	1224	407	8e-151	gp61 putative baseplate protein [ϕPLPE]
63	+	40465	41106	642	213	4e-44	gp62 [ϕPLPE]; P/BCHP
64	+	41110	42141	1032	343	3e-175	gp70 [*Vibrio* phage ICP1]; gp63 tail-fiber protein [ϕPLPE]
65	+	42143	42544	402	133	3e-39	gp00325 [*Vibrio* phage ICP1_2004_A]
67	+	42742	43278	537	178	3e-70	gp26T [*Vibrio* phage VP16T]; putative secretion activator protein
68	+	43278	43787	510	169	4e-8	gp0023 [*Thalassomonas* phage BA3]
69	+	43784	44212	429	142	3e-6	gp67 [ϕPLPE]
70	+	44215	44478	264	87	7e-12	gp68 [ϕPLPE]

[a] Generally listed are the closest phage (if any) homologs of identifiable function, followed by identifiable bacterial homologs, and then phage/bacterial hypothetical proteins (if no function could be deduced) from BLASTp searches against the nr (or phage restricted) database using an *E*-value cutoff of $<10^{-4}$. Also included are additional hits/homologs of interest and hits to the Conserved Domain Database (CDD) with their cd/pfam/cl/PHA identifiers. (P/B)CHP = (phage/bacteria) conserved hypothetical protein.
