# Supplementary material for: Phage Morphology Recapitulates Phylogeny: The Comparative Genomics of a New Group of Myoviruses
Source: PLoS One. 2012 Jul 6;7(7):e40102. doi: 10.1371/journal.pone.0040102 (PMC3391216; doi:10.1371/journal.pone.0040102)
Supplement: Table S5 — Vibrio phage 138 ORFs with identifiable homologs/protein functions. (PDF) [file pone.0040102.s005.pdf]

Table S5. *Vibrio* phage 138 ORFs with identifiable homologs/protein functions.

ORF	Strand	Start	End	nt length	aa length	*E*-value	Homolog(s)/Function[a]
1	-	1	534	534	177	2e-57	putative exodeoxyribonuclease VIII [*Salmonella* phage epsilon34]; PHA02570 DexA
2	-	539	2815	2277	758	3e-49	DNA polymerase [*Burkholderia* phage BcepGomr]; cd05538 phage DNA PolB
3	-	2840	3724	885	294	2e-19	gp68 [*Burkholderia* phage BcepB1A]
5	-	3946	5127	1182	393	3e-36	gp67 [*Burkholderia* phage BcepB1A]; pfam10926 PCHP
6	-	5106	5930	825	274	6e-9	gp66 [*Burkholderia* phage BcepB1A]
9	+	6513	8435	1923	640	4e-112	putative helicase [*Vibrio* phage VP16T]; COG1896 predicted hydrolase
10	+	8466	9449	984	327	9e-42	NinC protein [Enterobacteria phage lambda]
11	+	9442	9924	483	160	1e-41	gp62 [*Burkholderia* phage BcepB1A]
12	+	9921	11204	1284	427	2e-107	Dmt [Enterobacteria phage P1]; cd00315 DNA methylase
13	+	11201	11902	702	233	2e-99	adenine-specific DNA methyltransferase [*Pseudoalteromonas* phage H105/1]; pfam01555
14	+	11942	12256	315	104	8e-9	gp14 [ϕPLPE]; putative TetR family transcriptional regulator; pfam00440
17	+	12721	12924	204	67	6e-10	gp0660 [*Thiomicrospira crunogena* XCL-2]
19	+	13546	16026	2481	826	2e-43	gp49 primase [*Burkholderia* phage BcepB1A]
21	+	16879	17526	648	215	5e-21	TerS [ϕPLPE]
22	+	17507	18895	1389	462	2e-123	TerL [*Burkholderia ambifaria* phage BcepF1]
23	+	18899	20368	1470	489	0	gp19 putative portal protein [ϕPLPE]
28	+	21425	22246	822	273	2e-93	gp25 putative head protein [ϕPLPE]
30	-	22535	23038	504	167	2e-33	putative helicase [*Vibrio* phage VP16T]; COG1896 predicted hydrolase
32	-	23524	23853	330	109	2e-5	gp26 [ϕPLPE]
33	+	23852	25102	1251	416	2e-77	gp27 putative head protein [ϕPLPE]
34	+	25084	25608	525	174	2e-22	gp28 [ϕPLPE]; P/BCHP
35	+	25614	26627	1014	337	1e-99	gp29 major capsid protein [ϕPLPE]
36	-	26665	27369	705	234	8e-22	gp100 [*Rhizobium* phage 16-3]; P/BCHP
38	+	27540	27863	324	107	3e-6	gp30 [ϕPLPE]
40	+	28548	28973	426	141	1e-35	gp41 [ϕPLPE]; P/BCHP
43	-	29684	30022	339	112	5e-41	gp0180 [*Vibrio* phage KVP40]
44	+	30102	30572	471	156	9e-29	gp42 [ϕPLPE]; P/BCHP
45	+	30573	30953	381	126	8e-34	gp43 [ϕPLPE]
46	+	30950	31486	537	178	6e-52	gp44 [ϕPLPE]
47	+	31519	32664	1146	381	2e-170	gp46 [ϕPLPE]; P/BCHP
48	+	32678	33091	414	137	2e-59	gp47 [ϕPLPE]
51	-	33459	34070	612	203	1e-19	gpHK24401 [Enterobacteria phage HK244]; P/BCHP
52	+	34152	34565	414	137	1e-13	gp48 [ϕPLPE]
53	+	34610	34732	123	40	1e-8	gp49 [ϕPLPE]

54	+	34734	36098	1365	454	5e-57	gp55 putative tail-fiber/lysozyme protein [ϕPLPE]
55	+	36108	36755	648	215	5e-76	gp56 [ϕPLPE]; P/BCHP
56	+	36752	37060	309	102	2e-29	gp57 [ϕPLPE]
57	+	37053	37907	855	284	2e-91	gp58 [ϕPLPE]; P/BCHP
58	+	38233	38931	699	232	1e-98	gp59 putative baseplate protein [ϕPLPE]
59	+	38928	39278	351	116	5e-53	gp60 [ϕPLPE]; P/BCHP
60	+	39271	40494	1224	407	2e-154	gp61 putative baseplate protein [ϕPLPE]
61	+	40491	41132	642	213	2e-48	gp62 [ϕPLPE]; P/BCHP
62	+	41136	42389	1254	417	0	gp35 [*Vibrio* phage Ch457]; many tail fibers
63	+	42389	42916	528	175	9e-48	putative tail fiber assembly protein [*Vibrio* phage K139]
65	+	43135	43599	465	154	2e-34	putative phage lysozyme [*Listonella* phage phiHSIC]
66	+	43596	44105	510	169	3e-11	gp0023 [*Thalassomonas* phage BA3]

[a] Generally listed are the closest phage (if any) homologs of identifiable function, followed by identifiable bacterial homologs, and then phage/bacterial hypothetical proteins (if no function could be deduced) from BLASTp searches against the nr (or phage restricted) database using an *E*-value cutoff of $<10^{-4}$. Also included are additional hits/homologs of interest and hits to the Conserved Domain Database (CDD) with their cd/pfam/cl/PHA identifiers. (P/B)CHP = (phage/bacteria) conserved hypothetical protein.
