# Supplementary material for: Phage Morphology Recapitulates Phylogeny: The Comparative Genomics of a New Group of Myoviruses
Source: PLoS One. 2012 Jul 6;7(7):e40102. doi: 10.1371/journal.pone.0040102 (PMC3391216; doi:10.1371/journal.pone.0040102)
Supplement: Table S1 — Aeromonas phage 56 ORFs with identifiable homologs/protein functions. (PDF) [file pone.0040102.s001.pdf]

Table S1. *Aeromonas* phage 56 ORFs with identifiable homologs/protein functions.

ORF	Strand	Start	End	nt length	aa length	*E*-value	Homolog(s)/Function[a]
4	-	689	1225	537	178	8e-24	gp19 [*Salmonella* phage SE1]; putative lysozyme [ϕPLPE]; cd00737 endolysin
8	-	2258	3043	786	261	1e-6	structural protein gp26 [ϕPLPE]
9	-	3036	3353	318	105	7e-25	gp58 [*Klebsiella* phage phiKO2]; P/BCHP
11	-	3640	4182	543	180	2e-8	Dam methylase [*Yersinia* phage PY100]; pfam05869
16	-	5026	5508	483	160	4e-16	single-strand DNA binding protein [*Yersinia* phage PY100]; cd04496
17	-	5508	6191	684	227	9e-54	exonuclease [Enterobacteria phage VT2phi_272]; cl09232 YqaJ viral recombinase
18	-	6188	7066	879	292	3e-103	RecT protein [Enterobacteria phage VT2phi_272]; pfam03837
23	+	8378	8734	357	118	4e-16	gp07412 [*Pseudomonas syringae pv. glycinea* str. B076]
24	+	8731	9483	753	250	1e-13	DnaA [*Lactobacillus* phage Lv-1]; many DNA replication; pfam09524 PCHP
25	+	9473	10057	585	194	2e-56	gp0037 [*Thalassomonas* phage BA3]
30	+	11506	11871	366	121	1e-17	Nin B [Enterobacteria phage HK022]; pfam05772
33	+	12474	12725	252	83	1e-8	gp58 [*Acinetobacter* phage AB1]
37	+	13421	13792	372	123	7e-9	gp4530 [*Desulfatibacillum alkenivorans* AK-01]
38	+	13789	14505	717	238	3e-60	Ant [*Cronobacter* phage ENT47670]; pfam03374
39	+	14505	16097	1593	530	2e-15	gp0002 [*Thalassomonas* phage BA3]; putative methylase [*Methyloversatilis universalis* FAM5]
40	+	16090	16701	612	203	6e-42	NinG [*Pseudomonas* phage PAJU2]; pfam05766
43	+	17375	17650	276	91	4e-15	gp04 [*Cronobacter* phage ES2]
47	+	18566	19156	591	196	1e-58	TerS [Enterobacteria phage phiV10]
48	+	19140	20606	1467	488	6e-61	TerL [*Listeria* phage B054]
49	+	20786	21364	579	192	5e-37	gp04 [Enterobacteria phage K1ind3]; P/BCHP
50	+	21374	22924	1551	516	6e-111	gp30 [*Acinetobacter* phage AB1]; gp19 putative portal protein [ϕPLPE]
51	+	22924	23700	777	258	3e-53	putative head protein [*Xanthomonas* phage OP2]; many prophage heads
55	-	24860	25123	264	87	4e-15	gp2072 [*Vibrio cholerae* NCTC 8457]; BCHP
59	+	28654	28998	345	114	1e-6	gp13 [*Acinetobacter* phage AB1]; P/BCHP
60	+	29002	29397	396	131	2e-26	gp6 [*Xanthomonas* phage OP2]; P/BCHP
62	+	29574	30146	573	190	1e-7	gp20 [*Burkholderia* phage BcepB1A]; P/BCHP
63	+	30150	30524	375	124	5e-21	gp4 [*Xanthomonas* phage OP2]; P/BCHP
64	+	30521	31039	519	172	2e-6	gp3 [*Xanthomonas* phage OP2]; P/BCHP
65	+	31050	32528	1479	492	2e-94	gp2 [*Xanthomonas* phage OP2]; P/BCHP
66	+	32541	32978	438	145	4e-22	gp1 [*Xanthomonas* phage OP2]; P/BCHP
67	+	33059	33448	390	129	4e-6	gp01 [*Acinetobacter* phage AB1]
68	+	33484	33660	177	58	2e-8	gp15' E' [*Burkholderia* phage BcepB1A]

69	+	33669	35357	1689	562	2e-21	gp84 [*Acinetobacter* phage AB1]; gp55 putative tail-fiber/lysozyme protein [ϕPLPE]; PHA02564 virion
73	+	35948	36592	645	214	6e-14	gp83 [*Acinetobacter* phage AB1]; P/BCHP
74	+	36600	36899	300	99	2e-8	gp12 [*Burkholderia* phage BcepB1A]; P/BCHP
75	+	36914	37756	843	280	3e-20	gp82 [*Acinetobacter* phage AB1]; P/BCHP
76	+	37758	38390	633	210	6e-25	baseplate assembly protein [*Burkholderia* phage BcepNY3]
77	+	38390	38734	345	114	1e-18	gp80 [*Acinetobacter* phage AB1]; P/BCHP
78	+	38727	39308	582	193	8e-36	gp04 [Enterobacteria phage K1ind3]; P/BCHP
79	+	39353	40567	1215	404	4e-66	gp30 [*Xanthomonas* phage OP2] ; gp61 putative baseplate protein [ϕPLPE]
80	+	40564	41202	639	212	2e-27	gp29 [*Xanthomonas* phage OP2]; P/BCHP
81	+	41223	41945	723	240	3e-10	tail fiber protein [*Burkholderia* phage BcepNY3]
82	+	41972	42829	858	285	4e-40	tail fiber [Enterobacteria phage HK97]
83	+	42826	43434	609	202	9e-27	putative fiber assembly protein [Enterobacteria phage λ]; pfam02413 phage tail-fibers

[a] Generally listed are the closest phage (if any) homologs of identifiable function, followed by identifiable bacterial homologs, and then phage/bacterial hypothetical proteins (if no function could be deduced) from BLASTp searches against the nr (or phage restricted) database using an *E*-value cutoff of $<10^{-4}$. Also included are additional hits/homologs of interest and hits to the Conserved Domain Database (CDD) with their cd/pfam/cl/PHA identifiers. (P/B)CHP = (phage/bacteria) conserved hypothetical protein.
